# Biomimetic strategy for constructing *Clostridium thermocellum* cellulosomal operons in *Bacillus subtilis*

**DOI:** 10.1186/s13068-018-1151-7

**Published:** 2018-06-07

**Authors:** Jui-Jen Chang, Marimuthu Anandharaj, Cheng-Yu Ho, Kenji Tsuge, Tsung-Yu Tsai, Huei-Mien Ke, Yu-Ju Lin, Minh Dung Ha Tran, Wen-Hsiung Li, Chieh-Chen Huang

**Affiliations:** 1 Department of Medical Research, China Medical University Hospital, China Medical University, Taichung, 402 Taiwan; 2 Biodiversity Research Center, Academia Sinica, Taipei, 11529 Taiwan; 3 Molecular and Biological Agricultural Sciences Program, Taiwan International Graduate Program, National Chung Hsing University and Academia Sinica, Taipei, 11529 Taiwan; 4 Graduate Institute of Biotechnology, National Chung Hsing University, Taichung, 40227 Taiwan; 5 Department of Life Sciences, National Chung Hsing University, Taichung, 40227 Taiwan; 6 Institute for Advanced Biosciences, Keio University, 403-1 Nipponkoku, Daihoji, Tsuruoka, Yamagata 997-0017 Japan; 7 Biotechnology Center, National Chung Hsing University, Taichung, 40227 Taiwan; 8 Department of Ecology and Evolution, University of Chicago, Chicago, IL 60637 USA; 9 Innovation and Development Center of Sustainable Agriculture, National Chung Hsing University, Taichung, 40227 Taiwan

**Keywords:** Cellulosome, *Bacillus subtilis*, *Clostridium thermocellum*, Biomimetic strategy, Biomimetic operon

## Abstract

**Background:**

Enzymatic conversion of lignocellulosic biomass into soluble sugars is a major bottleneck in the plant biomass utilization. Several anaerobic organisms cope these issues via multiple-enzyme complex system so called ‘cellulosome’. Hence, we proposed a “biomimic operon” concept for making an artificial cellulosome which can be used as a promising tool for the expression of cellulosomal enzymes in *Bacillus subtilis*.

**Results:**

According to the proteomic analysis of *Clostridium thermocellum* ATCC27405 induced by Avicel or cellobiose, we selected eight highly expressed cellulosomal genes including a scaffoldin protein gene (*cipA*), a cell-surface anchor gene (*sdbA*), two exoglucanase genes (*celK* and *celS*), two endoglucanase genes (*celA* and *celR*), and two xylanase genes (*xynC* and *xynZ*). Arranging these eight genes in two different orders, we constructed two different polycistronic operons using the ordered gene assembly in *Bacillus* method. This is the first study to express the whole CipA along with cellulolytic enzymes in *B. subtilis*. Each operon was successfully expressed in *B. subtilis* RM125, and the protein complex assembly, cellulose-binding ability, thermostability, and cellulolytic activity were demonstrated. The operon with a higher xylanase activity showed greater saccharification on complex cellulosic substrates such as Napier grass than the other operon.

**Conclusions:**

In this study, a strategy for constructing an efficient cellulosome system was developed and two different artificial cellulosomal operons were constructed. Both operons could efficiently express the cellulosomal enzymes and exhibited cellulose saccharification. This strategy can be applied to different industries with cellulose-containing materials, such as papermaking, biofuel, agricultural compost, mushroom cultivation, and waste processing industries.

**Electronic supplementary material:**

The online version of this article (10.1186/s13068-018-1151-7) contains supplementary material, which is available to authorized users.

## Background

Biological conversion of lignocellulosic biomass into fermentable sugars is a sustainable approach for the production of biofuels and biochemicals with reduced environmental impacts [1, 2]. However, the major bottleneck is the low cellulolytic efficiency in converting the recalcitrant crystalline cellulose [3]. The highly ordered crystalline cellulose is buried within the architecture of cross-linked hemicellulose matrix covered by lignin, making it inaccessible for cellulolytic enzymes [4, 5]. Various techniques have been used to reduce the recalcitrant nature of lignocellulosic biomass, including ammonia treatment, chemical hydrolysis, and steam explosion. These methods increase the costs and produce toxic byproducts, which can inhibit the microbial growth [6]. In nature, certain wood-degrading fungal species efficiently hydrolyze lignocellulosic biomass via releasing an enzyme cocktail. In recent years, a large number of studies have focused on the pretreatment of biomass using the cellulase cocktail comprised of natural or tailor-made microbial enzymes [7].

Although such a cellulase cocktail can efficiently degrade biomass, its large-scale application is still not practical. On the other hand, a large multi-enzyme complex, the so-called ‘cellulosome’, which consists of many cellulases and hemicellulases, and can efficiently degrade plant biomass, has been identified from several anaerobic, cellulolytic bacteria, e.g., *Clostridium thermocellum* [8–10]. Indeed, a cellulosome can have 50 times higher enzymatic activity than the free cellulase enzymes secreted by *Trichoderma longibrachiatum* [11]. The enzymatic subunits of a cellulosome are assembled as a multi-enzyme complex on a non-catalytic scaffolding protein (CipA) and the whole complex is attached on the bacterial cell surface by an anchoring protein (i.e., SdbA, OlpB, Orf2P) [12, 13].

However, as *C. thermocellum* has a slow growth rate and produces metabolic intermediates, it is not highly desirable for the industrial purpose [14]. Hence, several mini artificial cellulosomes have been constructed and demonstrated for their cellulose-degrading potential in industrial hosts such as *Escherichia coli* and yeast [15–19]. Since several researchers have developed novel approaches to use *Bacillus subtilis* as a host for efficient protein secretion [20–22]. *B. subtilis* is considered as a workhorse for the industrial production of various recombinant proteins, amino acids and fine chemicals [23]. Recently, researchers have expressed minicellulosomes in *B. subtilis* [24, 25]. Furthermore, a chimeric minicellulosome with the *EngB* endoglucanase and mini-CbpA1 scaffolding protein of *Clostridium cellulovorans* was expressed and its in vivo assembly was achieved in *B. subtilis* [26]. These artificial chimeric minicellulosomes containing modified scaffoldin proteins and several cellulase enzymes showed only moderate activity on the cellulosic substrate and cannot achieve the efficiency of native cellulosomes [27].

The purpose of this study was to mimic the native cellulosome of *C. thermocellum*. From the expression profiles of *C. thermocellum* cultured with crystalline cellulose or cellobiose as the sole carbon source [28], we selected eight dominantly expressed cellulosomal genes and transformed them into in *B. subtilis.* Using OGAB (the ordered gene assembly in *B. subtilis*) method [29], we constructed two types of “biomimetic operons” each of which encoded an artificial multi-enzyme complex. These operons mimic the overall architecture of native cellulosomes in *C*. *thermocellum* and facilitate the enzyme synergism in *B. subtilis*. Finally, the cellulolytic efficiency of biomimetic cellulosomes was demonstrated using raw Napier grass.

## Methods

### Strains, media, and reagents

*Escherichia coli* DH5α (Real Biotech Corporation, Taiwan) and *B. subtilis* BUSY 9797 were used for genetic manipulations. Restriction modification-deficient mutant strain *B. subtilis* RM125 and protease-mutant *B. subtilis* WB800 were used as hosts for expressing various cellulosomal complexes [30]. All the bacterial strains used were cultured in Luria–Bertani (LB) medium (Difco Laboratories, Detroit, MI) supplemented with ampicillin 50 µg/ml (for *E. coli*) or tetracycline 10 µg/ml (for *B. subtilis*) and incubated at 37 °C for 24 h. The restriction enzymes were purchased from New England Biolabs and all the chemicals were purchased from Sigma-Aldrich (St. Louis, MO, USA) unless otherwise stated. The detailed information of each cellulosomal gene used in this study was provided in Additional file 1: Table S1. The cellulosomal genes were amplified from *C. thermocellum* ATCC 27405 genomic DNA using gene-specific primers (Additional file 1: Table S2).

### Biomimetic strategy for operon construction

The genes encoded for the cellulosomal complex subunits including CipA and other cellulase enzymes were amplified by PCR using the KOD-Plus Kit (TOYOBO CO., LTD., Japan). The amplified cellulosomal gene sequences were checked for its correctness using specific primer sets (Additional file 1: Table S3). The amplified PCR products were cloned into plasmid pCR-XL-TOPO, using the TOPO XL PCR Cloning Kit (Invitrogen, CA) and introduced into *E. coli* DH5α. The plasmids were purified using Qiagen Plasmid Midi Kit (Qiagen, CA, USA) and digested with specific restriction enzymes to prepare DNA fragments for further gene assembly. An *E. coli*/*B. subtilis* shuttle vector, pGETS118, was used to clone the DNA fragments in the designated order [31]. Eight cellulosomal gene fragments were ligated with a pGETS118 vector in designated order using the OGAB method for multiple-gene assembly in one step [22]. Briefly, equal molar DNA fragments of *CipA*, *CelK*, *CelS*, *CelR*, *CelA*, *XynC*, *XynZ*, and *SdbA* were mixed with pGETS118 vector, and the ligation was carried out at 16 °C for 30 min using Takara Ligation Kit. Based on the expression profile of *C. thermocellum* grown in cellobiose or Avicel, we designed two different operons: Type I: *CipA*–*CelK*–*CelS*–*CelR*–*SdbA*–*CelA*–*XynC*–*XynZ*; and Type II: *CipA*–*XynZ*–*XynC*–*CelA*–*SdbA*–*CelK*–*CelR*–*CelS*.

The copy number of pGETS118 vector in *B. subtilis* tends to be close to one but can be increased by 1 mM isopropyl 1-thio-β-d-galactoside (IPTG). This vector includes a strong, thermo-inducible Pr promoter [31] and was co-operating with CI repressor. To improve the success rate of plasmid construction, a specific CI repressor-mutant *B. subtilis* strain BUSY 9797 was employed, which totally represses the expression of inserted genes. The resulting plasmids were re-purified and transformed into the expression host *B. subtilis* RM125.

### Sequence confirmation by gene deletion kit

The correctness of each construct was confirmed by gene sequencing. However, the sequencing of *CipA* was difficult due to the multiple tandem repeats. This problem was resolved using Deletion Kit for Kilo-Sequencing Kit, Takara, which facilitates the sequencing of long DNA fragments by inserting the gene into a pUC-related vector. The gene deletion and sequencing was conducted according to manufacturer’s protocol.

### Quantitative PCR analysis

Bacterial cells were cultured in LB broth at 37 °C with 200 rpm for 16 h. The template mRNA was purified from *B. subtilis* cells using RNeasy Protect Mini Kits (High Pure RNA Isolation Kit, Roche). The cDNA synthesis was performed using a reverse transcription kit (SuperScript™ II Kit, Invitrogen). The relative quantification of each gene was carried out using SYBR green I RT-PCR Kit (Roche 480 SYBR green I master, Roche) and gene-specific primer sets (the amplicon size was around 113–137 bp) (Additional file 1: Table S3), according to manufacturer’s protocol. The standard template plasmids were prepared and quantitatively determined by a spectrophotometer with Em485nm Ex/525nm (SpectraMax M2, Molecular Devices, CA, USA) with SYBR green staining. Standard curves were generated for each primer pair to estimate their amplification efficiency using the LightCycler software (LightCycler 480, Roche), and the quantitative PCR data were accordingly adjusted for use in subsequent analysis.

### Cellulosome complex purification and analysis

Three different strategies were employed to purify the cellulosomal proteins and cellulosome complex for further analysis. First, *B. subtilis* clones were cultured at 37 °C for 20 h and supernatant was harvested by centrifugation at 8000*g* for 10 min at 4 °C. For quantitative enzyme assays and zymogram analysis, the culture supernatant was filtered through 0.2-mm membrane filter (Sartorius, Germany) and condensed 25-folds using Vivaspin 20 (10-kDa cutoff) system (GE Healthcare). The condensed supernatant was then dissolved in 1 mM CaCl_2_ and 10 mM DTT-containing buffer and prepared without heat treatment. Second, 20 ml condensed supernatant was mixed with 1 g of Avicel–cellulose and incubated at 4 °C for 16 h. Then the cellulose pellet was collected by centrifugation and washed twice with phosphate buffer. Proteins adsorbed onto the cellulose pellet (cellulosome) were eluted by 20 ml 1% triethylamine, and condensed 100-fold using Vivaspin 20 (10-kDa cutoff) system before sodium dodecyl sulfate-polyacrylamide gel electrophoresis (SDS-PAGE) analysis. Third, heat treatment was employed as a protein purification strategy. Briefly, the condensed supernatant was treated at 70 °C for 30 min and denatured non-thermostable proteins were removed by the centrifugation at 12,000 rpm. Then the collected thermostable sample was analyzed by western blot using a primary antibody against CipA protein.

### Quantitative assays for enzyme activity

Enzyme activity was performed using 25-fold condensed culture supernatant of *B. subtilis* clones. Total glucanase activity was determined by mixing 50 µl of supernatant with 50 µl of buffer solution [50 mM 4-methylumbelliferyl-β-d-cellobiopyranoside (MUC), 50 mM sodium acetate, pH 3]. The enzyme activity of released 4-methylumbelliferone (MU) was measured in fluorescence units (FU) by fluorescent intensity reader (SpectraMax M2, Molecular Devices, USA). The endoglucanases were assayed by 1% (v/w) dye-CMC containing 50 mM Sodium acetate at pH5.0 and incubated at 60 °C for 16 h. Azurin dye liberated from dye-CMC was measured photometrically at 590 nm. Similarly, exoglucanase and xylanase activities were determined by mixing 50 µl of supernatant with 50 µl of a buffer solution containing either 2% phosphoric acid-swollen cellulose (PASC) or 2% xylan as a substrate. For the quality assay of glucanase activity, enzymes were incubated in 50 mM sodium acetate buffer at the same pH, temperature and time. The amount of reducing sugar and total soluble sugar released from the insoluble substrate and insoluble sugar was measured using the Somogyi–Nelson method by measuring the absorption at 520 nm.

### Gel electrophoresis and zymogram

SDS-PAGE was performed on a gel containing 5–15% (w/v) acrylamide and 0.1% SDS (w/v), using tris/glycine buffer system. The gel was submerged into the MUC solution (0.2 mg/ml MUC, 50 mM NaOAc pH5.0) at 60 °C for 30 min to detect glucanase activity. Zymogram analysis for xylanase and endoglucanase was performed using CMC and birch wood xylan (Sigma) as a substrate. To prepare substrate gel (11 × 12.5 cm), 500 mg of xylan or 1 g of CMC was dissolved in 100 ml of deionized water and mixed with 2% agarose. The mixture was autoclaved and then poured into Petri dish. After solidifying, the substrate gel was carefully overlaid with a protein gel. The gels were smoothed to remove bubbles, wrapped in plastic, and incubated at 60 °C overnight for enzyme reaction. Then gels were separated and stained with Coomassie brilliant blue. Substrate gels containing birch wood xylan or CMC were immersed in 1 mg/ml conge red for 30–60 min and de-stained with 1 M NaCl for 10–60 min. The yellow bands appeared against dark-red background was considered as a positive result.

### Immunofluorescence microscopy

The anchoring efficiency of SdbA on *B. subtilis* cell surface was demonstrated by designing a plasmid containing SdbA fused with RFP and HA tag (pHT254-RFP-HA-SdbA) for fluorescence detection. The plasmid was transformed into *B. subtilis* strain following the method of Zhang et al. [32]. To conduct the immunoblotting, bacterial cells expressing anchoring protein was collected (A_600_ of 3.0) and washed several times with PBS (pH 7.4). Then cells were resuspended in 4% paraformaldehyde (PFA) and incubated for 30–60 min at 4 °C. Cells were centrifuged and washed with PBS buffer to remove the excess PFA and resuspended in 500 µl of Bovine serum albumin (BSA, 1 mg/ml in PBS) containing 0.5 µg of anti-HA primary antibody (Biotools, Taiwan) and incubated for 3 h. Then cells were harvested by centrifugation and resuspended in 500 µl of PBS containing secondary antibody conjugated with DyLight™405 fluorophores (Jackson Immunoresearch, USA) and incubated for 1 h. After the incubation cells were washed three times, 2 µl of cell suspension was fixed in a slide and observed under Leica TCS SP5 II confocal microscopy (Wetzlar, Germany).

### Biomass saccharification

To demonstrate the cellulolytic efficiency of *B. subtilis* clones, Avicel, filter paper, and Napier grass were used. Briefly, 25-fold condensed culture supernatant with cellulosome complexes were harvested as an enzyme cocktail for cellulolytic reaction and mixed with Avicel or filter paper and incubated at 60 °C for 48 h. On the other hand, Napier grass saccharification was conducted by inoculating the OD 10 cells of three *B. subtilis* clones (Type I, Type II, and Control) in LB medium supplemented with 2% Napier grass and cultured at 42 °C with 200 rpm for several days. The nutrition of LB medium and the residual sugar in Napier grass supported *B. subtilis* growth and cellulosomal enzyme production. The amount of reducing sugar and total soluble sugar released from the insoluble substrate was measured using the Somogyi–Nelson method by measuring the absorption at 520 nm.

## Results

### Construction and confirmation of “biomimetic operons”

**Fig. 1:**
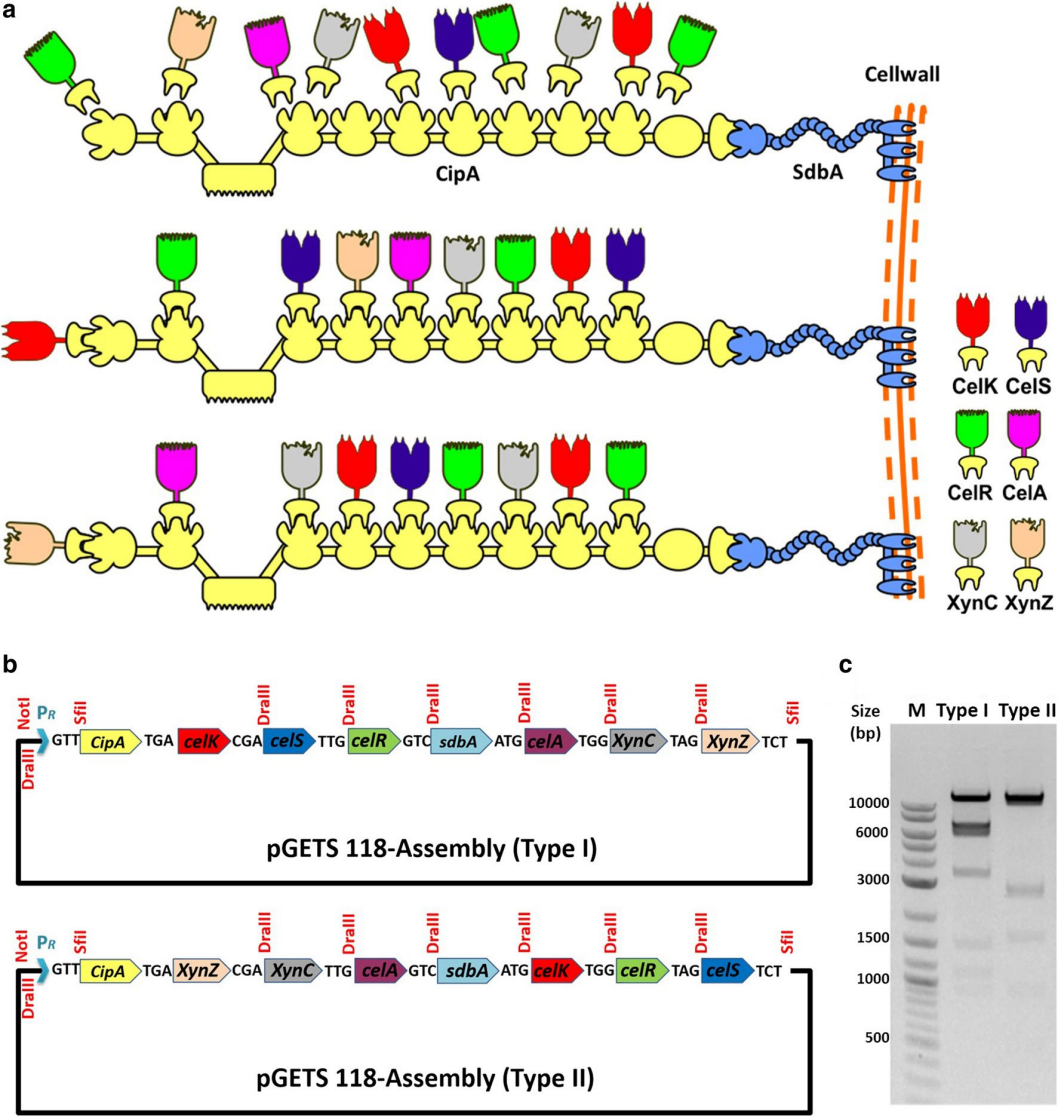
Schematic diagrams of a biomimetic cellulosome and a biomimetic operon. **a** Schematic representation of a biomimetic cellulosome and assembly of cellulosomal subunits on the cell surface. The cell-surface anchor (*sdbA*, shown in blue) is attached on the cell membrane. The scaffolding protein (CipA, shown in yellow) is composed primarily of nine copies of specific Type I cohesin and Type III carbohydrate-binding modules (CBM). **b** Construction of biomimetic polycistronic operons, Type I and Type II in pGETS 118 vector, restriction enzymes were given in red color. **c** The confirmation of gene assembly pattern by EcoRI Restriction digestion

Based on the proteome-wide expression analysis on cellobiose or Avicel, we constructed Type I and Type II “biomimetic operons”, respectively. The top eight cellulosomal genes from each expression profile were selected, including a scaffoldin protein gene (*cipA*) to express as a framework of cellulosome, two exoglucanase genes (*celK* and *celS*), two endoglucanase genes (*celA* and *celR*), two xylanase genes (*xynC* and *xynZ*) and a small cell-surface anchor (*sdbA*) gene. The selected genes were amplified from the *C. thermocellum* ATCC 27405 genome. The amplified cellulosomal genes were assembled and expressed as a biomimetic cellulosome (Fig. 1a). Each cellulosomal gene was amplified from the *C. thermocellum* genome along with 16-bp native ribosomal-binding site (RBS) and we have demonstrated the expression of each gene in *B. subtilis*, thus confirming that the RBS from *C. thermocellum* also works in *B. subtilis.* The sequencing results confirmed that the amplified cellulosomal genes were identical to the original sequences of *C. thermocellum* ATCC 27405, except CipA. Since the native CipA sequence was long (5562 bp) with many tandem repeats, nine different primer pairs were used for the sequence confirmation. The sequences of CipA were further confirmed by Gene Deletion Kit for Kilo-Sequencing. The amplified CipA had A→G base shift at the 1473rd position but the amino acid sequences were the same as the native CipA. This is the first study to amplify the whole native CipA from the genome of *C. thermocellum* and express it in *B. subtilis*.

pGETS118 vector was used as the backbone for the construction of biomimetic operon. Briefly, the vector was ligated with eight cellulosomal gene fragments in a pre-designated order using the OGAB method. A strong Pr promoter was used as the sole promoter to drive the entire polycistronic operon. To mimic the native cellulosome of *C. thermocellum*, the cellulosomal genes were assembled in two different designated orders (Type I and Type II), based on the expression profile of *C. thermocellum* grown in cellobiose or cellulose, respectively. The pGETS 118-Type I (38 kb) assembly containing the cellulosomal genes in the following order *cipA*–*celK*–*celS*–*celR*–*sdbA*–*celA*–*xynC*–*xynZ* (Fig. 1b). The gene assembly was done in *B. subtilis* 9797 and verified by *Eco*RI digestion, which yielded nine fragments with 14,465, 7442, 6162, 2982, 1540, 1285, 1151, 965, and 68 bp, respectively (Fig. 1c). Similarly, the Type II biomimetic operon was assembled according to the expression profile of *C. thermocellum* grown in cellulose. The pGETS 118-Type II plasmid includes cellulosomal genes in the following order: *cipA*–*xynZ*–*xynC*–*celA*–*sdbA*–*celK*–*celR*–*celS* (Fig. 1b). The pGETS 118-Type II plasmid was verified by *Eco*RI restriction enzyme digestion and generated eight fragments with 15,750, 11,549, 2846, 2685, 1655, 965, 543, and 68 bp, respectively (Fig. 1c). The restriction enzyme map confirmed the arrangements of cellulosomal subunits with the designated order in the Type I and Type II pGETS 118 vectors.

To confirm that the biomimetic operons were driven by the sole Pr promoter, two *B. subtilis* clones (Type I and Type II) were grown in LB medium for 16 h for gene transcription assay. Total RNA was extracted from each of these two clones and an absolute qRT-PCR analysis was conducted with gene-specific primer pairs using the SYBR green detection method. The data indicated that all of the eight heterologous genes, *cipA*, *celK*, *celS*, *celR*, *sdbA*, *celA*, *xynC*, and *xynZ*, in both Type I and Type II operons were expressed (data not shown).

### Expression of cellulosomal proteins

**Fig. 2:**
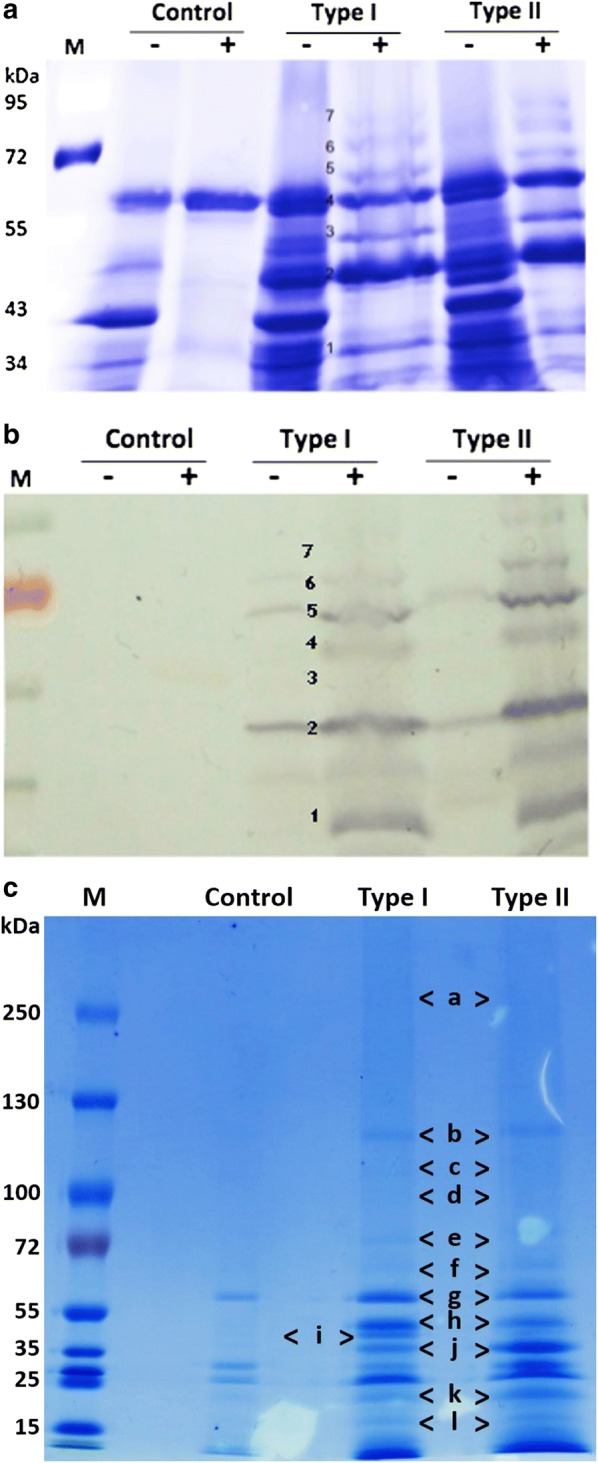
Expression and assembly pattern analysis of “biomimetic cellulosomes”. **a** SDS-PAGE and **b** western blot analysis of culture supernatant (heat-treated at 70 °C) of CipA. **c** SDS-PAGE analysis of cellulosomal proteins purified using cellulose adsorption via the scaffoldin protein CipA with CBM (+, heat treatment at 70 °C for 30 min; −, without heat treatment; M, protein marker. The expressed proteins were marked using alphabets, and/or numbers

The expression and secretion of these heterologous proteins were confirmed by comparing the culture supernatants of the Type I, Type II and control (pGETS 118 vector only) strains. Two strategies were employed to enrich the protein profile of the expressed cellulosome. First, a heat treatment at 70 °C for 30 min was applied before protein purification, because the enzymes of *C. thermocellum* display better cellulase activity at 70 °C [33, 34]. SDS-PAGE data showed clear cellulosomal protein bands from the supernatants of the Type I and Type II strains, but not the control strain (Fig. 2a, b). Furthermore, seven dominant protein bands were observed on SDS-PAGE and identified as the scaffoldin protein CipA via protein mass spectrometry assay, and was further confirmed by western blot analysis using epitope antibody against CipA (Additional file 1: Fig. S1). Protein bands were further confirmed by Protein Mass spectrometry analysis using the *C. thermocellum* protein database as a reference in the Mascot database (Matrix Science, MA, USA). The top five hits revealed the presence of CipA along with other cellulase enzymes such as CelK, CelA, CelR, XynC, and XynZ. These cellulosomal proteins were successfully expressed and secreted by the Type I and Type II strains; some of the truncated CipA might be due to disruption by the heat treatment.

Second, the cellulosomal proteins were purified using microcrystalline cellulose, since CipA contains a CBM domain. The cellulosome complex was adsorbed into the cellulose pellet for 16 h at 4 °C and adsorbed proteins were eluted using 1% triethylamine. Then the purified proteins were analyzed on SDS-PAGE and the results showed several distinct bands. Based on the molecular weights, bands a, d, e, and h were identified as CipA (192.8 kDa), CelK (100.6 kDa), XynZ (92.2 kDa), and CelA (52.5 kDa) (Fig. 2c). Band f might contain both CelS and CelR because their molecular weights are similar (83.5 kDa vs. 82 kDa). Although band g also appeared in the control strain, the intensity of the band was higher in both Type I and Type II clones, so this band might also contain XynC (69.5 kDa). These data indicated that all of the cellulosomal proteins, except SdbA, were expressed and secreted by *B. subtilis* Type I and Type II clones, and they were assembled with CipA, which harbors a CBM domain. The anchoring protein SdbA band was not observed in the supernatants because they might have anchored on the cell wall. In addition to the major bands, several other bands such as b, c, h, i, j, and k with molecular weights of ~ 125, 110, 40, 35, 20, and 15 kDa, respectively, were also observed. These fragments were identified as partial fragments of CipA by protein Mass Spectrometry analysis (Additional file 1: Fig. S2) and indicated that CipA might be disrupted by the protease digestion of the heterologous *B. subtilis* host.

### Assembly of the artificial multi-enzyme complex

**Fig. 3:**
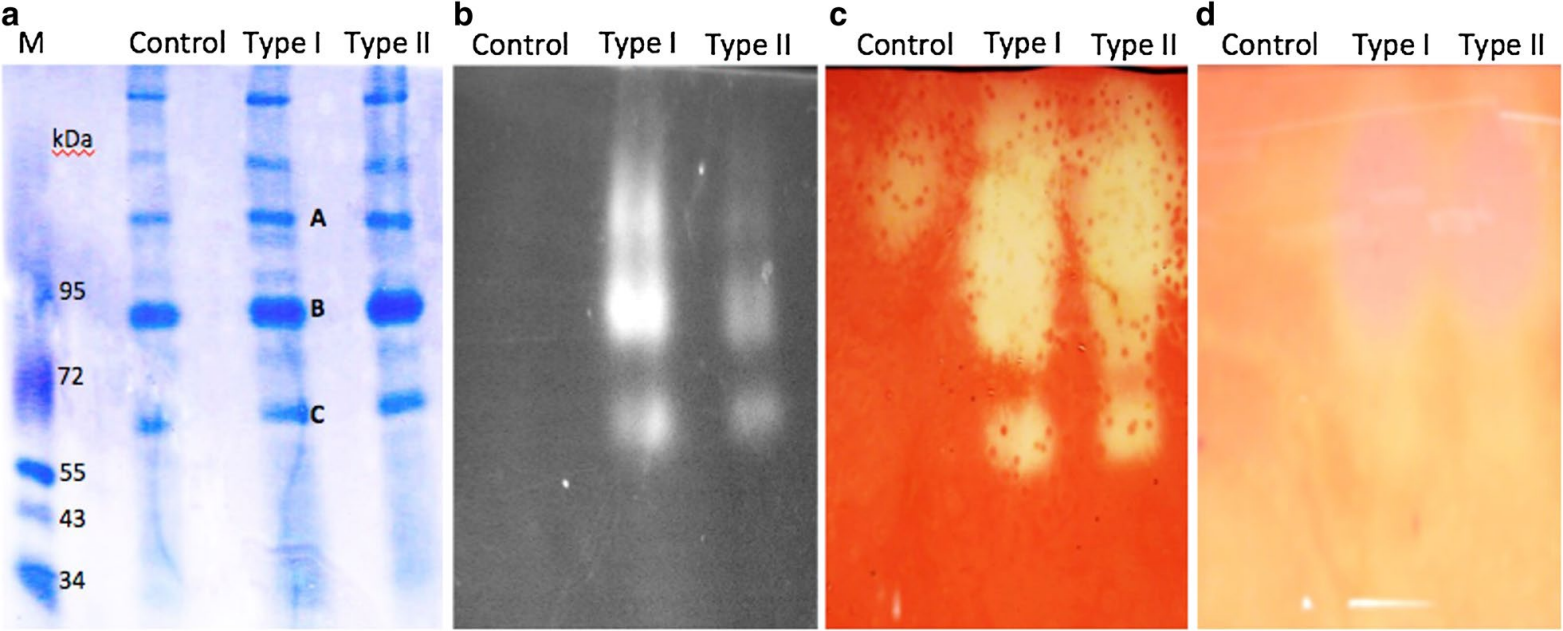
Zymogram analysis of cellulosomal complex. Zymogram analysis was conducted using the supernatants of the Type I, Type II, and Control strains. **a** A 5–15% native PAGE analysis stained with coomassie brilliant blue R-250. **b** Zymogram of total glucanase using MUC as substrate. **c** Zymogram of endoglucanase with dye-CMC as substrate. **d** Zymogram of xylanase using xylan as substrate

The interaction of cellulosomal enzymes (containing a Type I dockerin domain) with CipA (containing nine Type I cohesins) and their multiple-protein complex assembly was confirmed by a gradient native PAGE gel electrophoresis (Fig. 3a). In native PAGE, the molecular weight of a protein might be different with their expected molecular weight due to different charges or isoforms of an enzyme [35]. Total glucanase activity of the cellulosome complex was confirmed by a strong smeared band in zymogram, as the Type I strain had a strong smear of the band compared with the Type II strain (Fig. 3b). Endoglucanases in the cellulosome complex showed a smear of bands in both the Type I and Type II strains against the dark stained background (Fig. 3c) and these data suggested that the two strains had similar expression levels. Moreover, a weak smear was observed in the control strain, which might be due to the endogenous endoglucanase activity of *B. subtilis* [36, 37]. However, these endogenous cellulases might not interfere with the cellulosome complex assembly, because *B. subtilis* cellulases might not contain any dockerin domain to interact with CipA protein. Also the cellulosomal proteins of *C. thermocellum* have high affinity cohesin–dockerin interactions and will not interact with other nonspecific proteins. Similarly, the xylanase activity of the cellulosome complex was determined by zymogram analysis using xylan as the substrate and both Type I and Type II strains showed strong xylanase activities (Fig. 3d).

Furthermore, three dominant bands (A, B, and C) with enzyme activity were excised from the native PAGE and the corresponding proteins were identified using mass spectrometry analysis. Except CelS and SdbA, other cellulosomal proteins including CelK, XynZ, CelR, XynC, and CelA were identified in each band, suggesting that seven cellulosomal subunits could assemble as protein complexes of different sizes due to the various truncated forms of scaffoldin protein CipA. Therefore, the proposed polycistronic operon strategy is a reliable approach for regulating the cellulosomal subunits in a cellulosome complex.

### Functional assays of cellulolytic enzymes

**Fig. 4:**
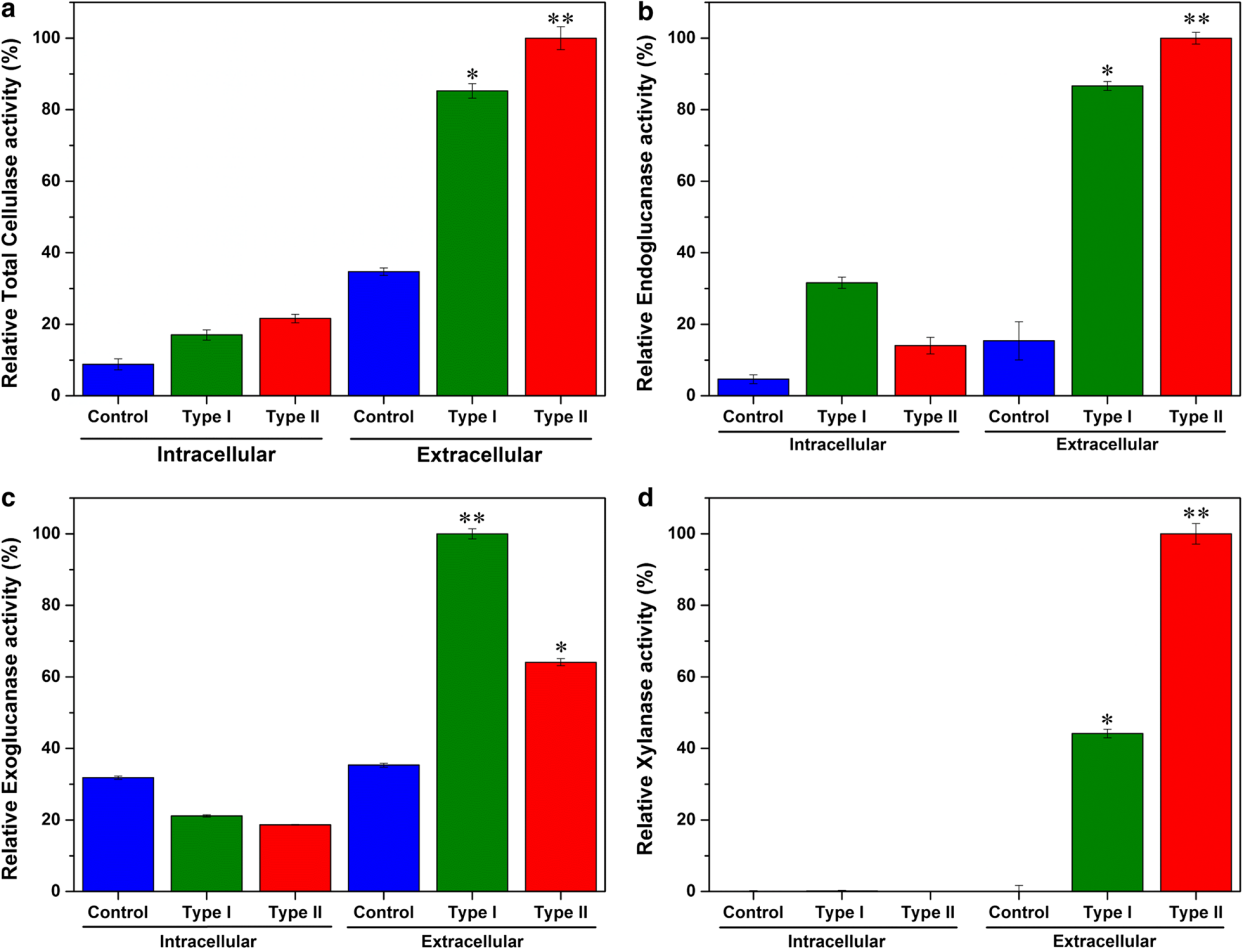
Specific and quantitative enzyme assays for biomimetic cellulosomes. To quantify the enzyme activities of cellulosomal enzymes, both supernatant and intracellular materials were derived from the Type I, Type II, and Control strains and were used for enzyme assay. **a** Total glucanase activity assay using MUC as substrate. **b** Relative endoglucanase activity using dye-CMC as substrate. **c** Relative exoglucanase activity using PASC as substrate. **d** Relative xylanase activity using xylan as substrate. Results are expresed as Mean ± SD (*n* = 3). ^∗^*P* ≤ 0.05, ^∗∗^*P* ≤ 0.01

To quantify the variations in exoglucanase, endoglucanase, and xylanase enzyme activity, the Type I, Type II and control strains were cultured and harvested, and both supernatants and cell lysates were used for enzyme assays. The total glucanase activity was analyzed using MUC as substrate. Both the Type I and Type II strains exhibited highest activity than the control strain in supernatants and weak enzyme activity was observed in cell lysates (Fig. 4a). These data confirmed that both the Type I and Type II strains were successfully expressed and secreted out in the supernatant. The endoglucanase activity data of both the Type I and Type II strains were similar and higher than the control strain (Fig. 4b). Considerable variations in exoglucanase and xylanase activity were observed between the Type I and Type II strains (Fig. 4c, d). The Type I supernatant exhibited significantly higher exoglucanase activity compared with the Type II supernatant (Fig. 4c). The highest xylanase activity was observed in the supernatant of the Type II strain (Fig. 4d).

**Fig. 5:**
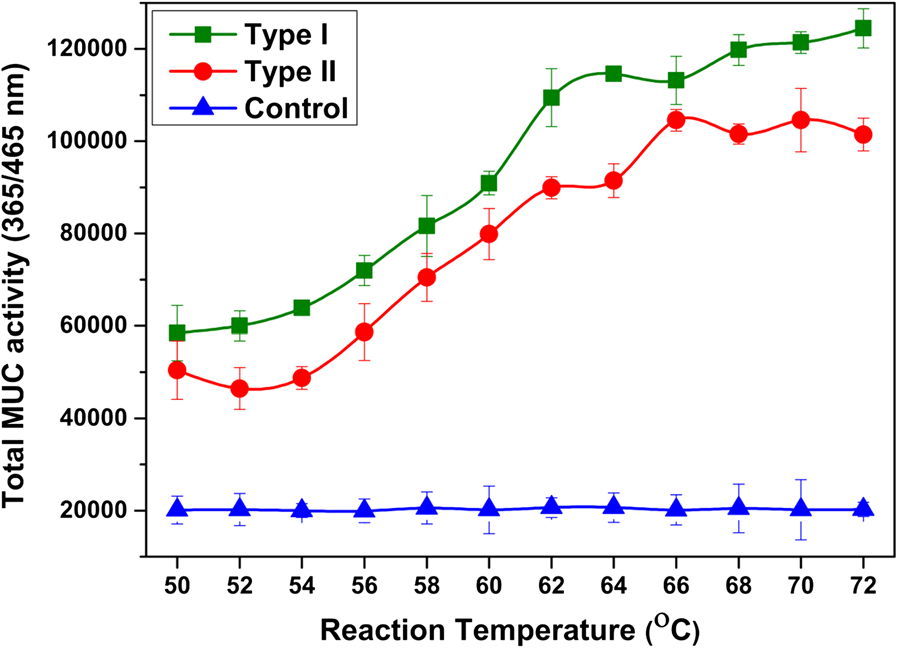
The total glucanase activity enzyme assay of cellulosomal proteins under different temperatures

The thermostability of the cellulosomal enzymes was determined by total glucanase activity assay using MUC as substrate. The data showed higher activity at 72 °C in the Type I and Type II strains than in the control (Fig. 5). Interestingly, the thermostability of our *B. subtilis* cellulosome was similar to that of the native cellulosome of different *C. thermocellum* strains [38, 39].

### Confirmation of SdbA anchoring efficiency

**Fig. 6:**
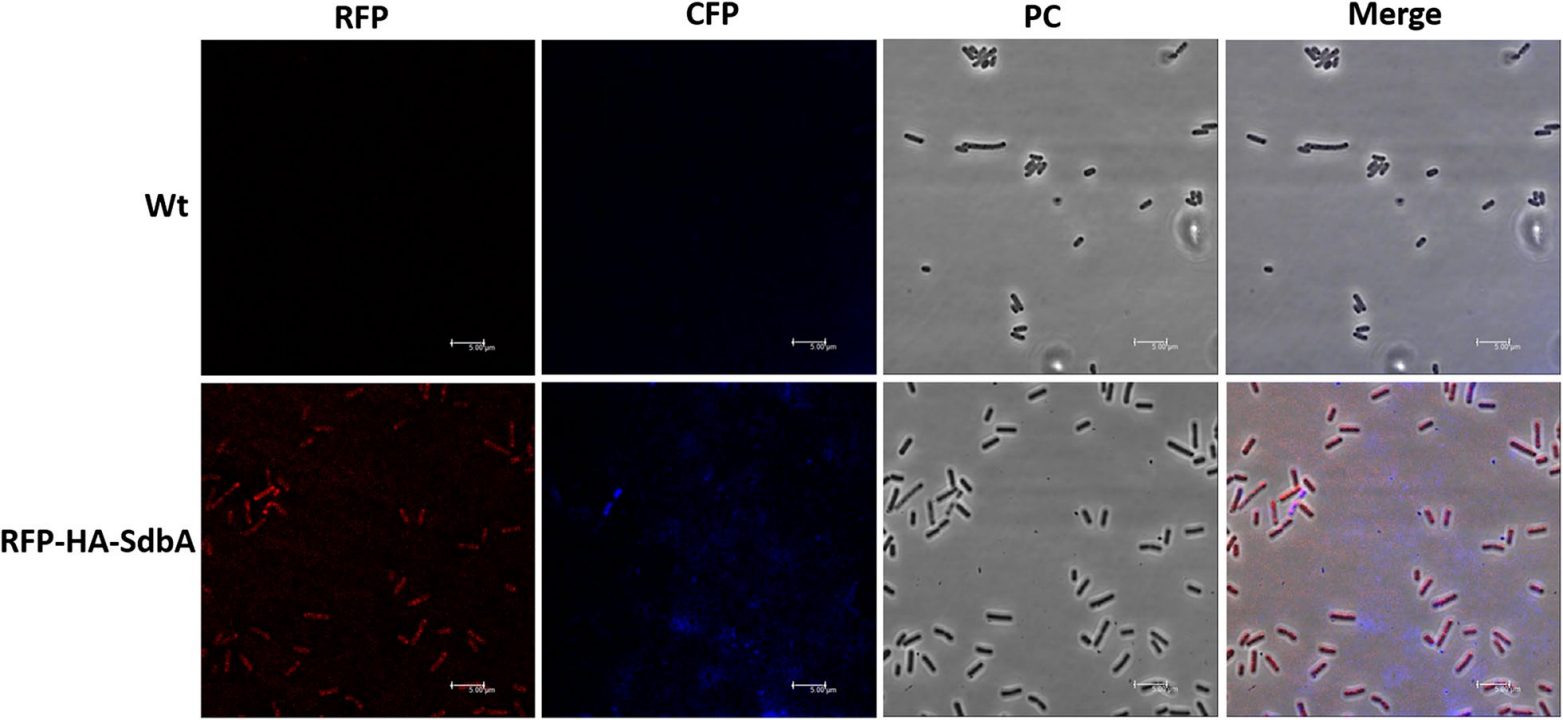
Immunofluorescence microscopic analysis of *B. subtilis*. The anchoring construct (pHT254-RFP-HA-SdbA) was expressed in *B. subtilis* and cell surface anchoring was detected using the anti-HA antibody as well as RFP signal. Wt: *B. subtilis* control strain without plasmid, RFP-HA-SdbA: *B. subtilis* engineered strain with pHT254-RFP-HA-SdbA

In our study, the S-layer anchoring protein (SdbA) was expressed in Type I, Type II and control strains, and it might be anchored on the cell wall and interact with the Type II cohesin of CipA with high affinity. To detect the Type II cohesin–dockerin interaction, an epitope antibody of CipA was used for whole cell immunofluorescence analysis. Previous studies found that the cellulosome might escape from the cell surface at different growth stages [40]. Hence, the Type I, Type II and control cells were harvested at different growth stages for analysis, and *C. thermocellum* ATCC 27405 was used as a positive control. No fluorescence signals were observed by fluorescence microscopy except in *C. thermocellum* ATCC 27405, and the absence of fluorescence signals in the three *B. subtilis* strains might be due to the endogenous protease activity of *B. subtilis.* Indeed, the anchoring efficiency was confirmed by employing the protease-mutant strain *B. subtilis* W800. To further confirm the anchoring efficiency, pHT254-RFP-HA-SdbA construct was expressed and weak RFP fluorescence signal was observed on the cell surface of *B. subtilis*. Similarly, the weak fluorescence signal was observed on immunostaining using an anti-HA antibody, which confirmed that SdbA had very weak anchoring efficiency on *B. subtilis* cell surface (Fig. 6).

### Functional assay by Napier grass degradation

**Fig. 7:**
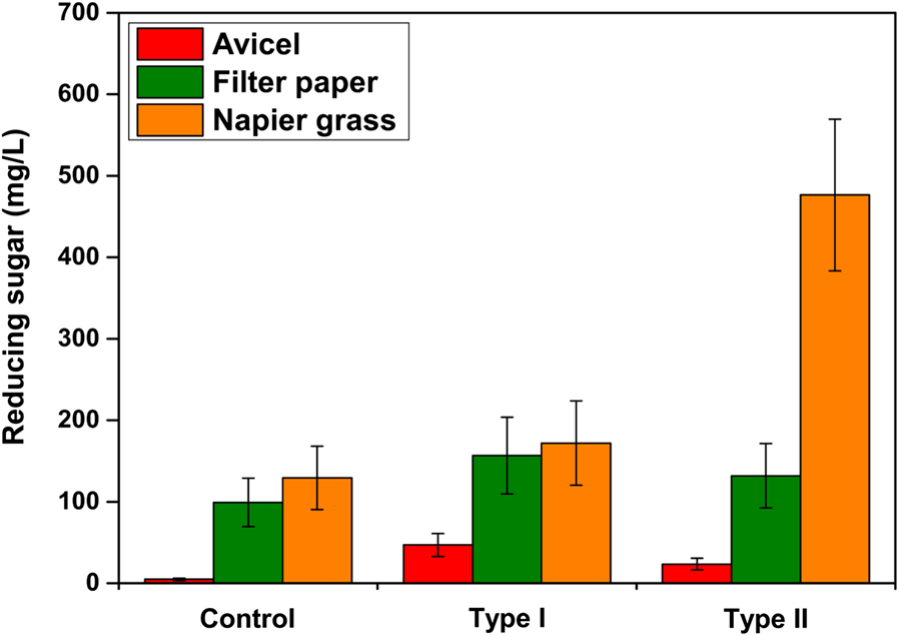
Biomass saccharification assays. Comparison of the cellulolytic ability of Type I, Type II, and Control strains. Avicel, filter paper, and Napier grass were used separately for accumulating reducing sugar assays

The cellulolytic efficiency of *B. subtilis* strains (Type I, Type II, and Control) on raw cellulosic materials was demonstrated using Avicel, filter paper, or Napier grass as substrate and reducing sugar release was determined. The supernatant of the Type I strain exhibited greater reducing sugar accumulation on Avicel and filter paper (46.91 and 156.81 mg/ml, respectively) than the Type II strain (Avicel 23.49 mg/ml and filter paper 131.53 mg/ml) (Fig. 7). On the other hand, no significant difference was observed on filter paper degradation between the Type I and Type II strains (Fig. 7).

The effect of cellulosome complex on raw biomass was demonstrated by the saccharification of Napier grass. The nutrition of LB medium and the residual sugar in Napier grass supported the *B. subtilis* growth and cellulosomal enzyme production. Interestingly, after 48 h of cultivation at 42 °C, Type II strain released two- to fourfold higher (476.69 mg/ml) reducing sugar than the Type I strain (171.99 mg/ml) and the control (129.26 mg/ml) (Fig. 7).

## Discussion

Lignocellulosic biomasses are abundant in nature and annually 60 billion tons of biomasses are produced throughout the world [41]. Sustainable utilization of these renewable biomasses to produce valuable products using microorganism is an ideal approach [42]. However, the major bottleneck in the utilization of biomass is to convert the crystalline cellulose and hemicellulose into soluble sugars due to its complex organization [43]. To date, ‘cellulosomes’ from anaerobic bacteria (e.g. *C. thermocellum*) has been considered as nature’s finest cellulolytic machinery. However, the industrial cultivation of *C. thermocellum* have several limitations; hence utilization of industrial hosts (i.e. Bacillus and yeast) for the expression of cellulosomal components is an ideal approach. In our study, we have selected *B. subtilis* as a host since *B. subtilis* is a generally regarded as safe (GRAS) organism by Food and Drug Administration (FDA) [44] and also provide several other benefits. *B. subtilis* is already known for the production of valuable metabolites, bioremediation and generation of bioenergy [45]. The complete genome sequence of *C. thermocellum* ATCC 27405 has been published (GenBank accession number: CP000568.1), which encodes ~ 50–60 cellulosomal enzymes [46]. The cellulolytic activity of *C. thermocellum* is regulated by either carbon source or growth rate (or both) and changes with respect to overall cellulosomal subunit profile at both the protein and the mRNA level [47, 48]. This observation agreed with the data of Gold and Martin [28], in which the expression profiles of cellulosomal subunits fluctuated with different substrates such as cellulose or cellobiose. Based on this, we have constructed two different “biomimic operons” such as Type I and Type II (Fig. 1b). We have successfully amplified the 5562-bp long CipA gene from *C. thermocellum* and used for the expression in *B. subtilis*. The total genomic G+C content of *C. thermocellum* and *B. subtilis* was found to be 40.34 and 44.36%, respectively. In addition, *C. thermocellum* had G+C content of 47.23, 33.94 and 39.85% at first, second and third codon positions, respectively. Similarly, the *B. subtilis* had G+C content of 52.10, 36.08 and 44.91% at first, second and third codon positions, respectively. These results showed that both of these bacterial species had almost a similar G+C content ratios. This is the first attempt to express the whole CipA along with cellulolytic enzyme on *B. subtilis*.

The cellulosomal components have been expressed and successfully assembled as a complex (Fig. 2). The protein mass spectrometry analysis of cellulosome complex reveals the presence of all the cellulosomal enzymes as well as the scaffoldin CipA; however, the anchoring protein (SdbA) was not detected by mass spectrometry. The data revealed that there is no SdbA protein on the complex because they might have anchored on the cell wall but the qPCR analysis showed that the SdbA was transcribed successfully. The immunofluorescence microscopic results also showed that there is very weak anchoring on *B. subtilis* cell surface, but the RFP fluorescence microscopic showed that the SdbA was anchored on the cell surface. This data revealed that the *C. thermocellum* SdbA may not be anchored efficiently on the *B. subtilis* cell surface or Type II cohesin–dockerin interaction may not be present efficiently; hence the secreted cellulosome was assembled as a complex in vitro rather than anchored on cell surface. Generally, cellulosomal proteins produced by *C. thermocellum* ATCC 27405 grown on cellulose was unaffected by oxygenic condition [33, 34]. On the other hand, our enzyme production host *B. subtilis* is strictly aerobic, so that the protein folding condition of SdbA might be different from that in the *C. thermocellum* and thus affects the anchoring efficiency in *B. subtilis*. To get efficient anchoring other kinds of S-layer anchor protein, such as Orf2p and OlpB, will be studied in the future.

The Type I and Type II strains were successfully expressed and secreted out the engineered cellulosomal enzymes. The endoglucanase activity of both Type I and Type II strains were similar (Fig. 4b). On the other hand, considerable variations in exoglucanase and xylanase activity were observed between the Type I and Type II strains (Fig. 4c, d). The Type I exhibited significantly higher exoglucanase activity then Type II (Fig. 4c) but displayed highest xylanase activity (Fig. 4d). These observations were correlated with the positions of genes in the polycistronic operon; the gene position on the operon is a key factor to regulate the enzyme ratio. For example, in Type I construct *CelK* and *CelS* were located near the Pr promoter, and they exhibited greater exoglucanase activity on PASC. On the other hand, in Type II construct, *XynZ* and *XynC* were located near the Pr promoter and demonstrated greater xylanase activity on xylan, compared with Type I.

Since the ultimate goal of artificial cellulosome is to completely solubilize the crystalline cellulose or raw biomass and produce higher concentrations of soluble sugars (i.e. cellobiose and glucose), sugar hydrolysis was performed using three different substrates. The Type I strain accumulated higher reducing sugar than the Type II on Avicel (Fig. 7). This was due to the strong exoglucanase activity of the Type I strain, which is in agreement with our previous enzyme activity assay. The above data were in accordance with the expression profile of cellulosomal subunits in *C. thermocellum* ATCC 27405 grown in cellulose [28], and the exoglucanase might play a crucial role in the degradation of crystalline cellulose. On the other hand, no significant difference was observed on filter paper degradation between the Type I and Type II strains (Fig. 7). In general, endoglucanases have the ability to invade the flabby filter paper and initiate its degradation but in our study both the Type I and Type II strains showed similar endoglucanase activities. Thus, we inferred that the exoglucanases play a key role in the degradation of stubborn crystalline cellulose.

The Type I and Type II strains were efficiently degrading the Napier grass and Type II strain released two- to fourfold higher reducing sugar than the Type I strain and the control (Fig. 7). These data confirmed the positional effects of cellulase genes in the polycistronic operon. The data indicated that xylanase may play a crucial role in the release of cellulose from the highly ordered architecture of insoluble Napier grass, which made a tight conjunction with hemicellulose and lignin polymers by xylan. On the other hand, Type I and control might not initiate cellulose degradation due to its low xylanase expression. In nature, the optimal ratio of different cellulases and the favorable timing for enzyme expression might be one of the critical factors for lignocellulosic biomass utilization. For instance, the time course profile for cellulolytic enzymes from ruminal microflora showed that the hydrolysis of complex lignocellulosic biomass (i.e. Napier grass) may occur through the ordered actions of xylanase and cellulase activities [49].

In this study, the ratio of enzyme compositions in the biomimetic cellulosomes affected substrate preference and this may provide new insights into raw cellulose degradation. In nature, plant materials such as Napier grass, cellulose-containing materials and microfibrils are associated with other components such as hemicellulose, lignin, and xylan, and also with some reducing sugars such as cellobiose. These simple sugar components might play the role of inducer in the initial stage of amorphogenesis while xylanase might be the key enzyme in enzymatic cellulose saccharification [50].

## Conclusion

In this study, a strategy for constructing an efficient cellulosome system was developed and two different artificial cellulosomal operons were constructed. Both operons could efficiently express the cellulosomal enzymes and exhibited cellulose saccharification. The enzyme expression levels of the eight selected genes depended on their positions on the polycistronic operon. Thus, for digesting different kinds of cellulosic substrates, the cellulosomal subunits were arranged in different orders and this was important in the design of a “biomimetic operon”. This strategy can be applied to different industries with cellulose-containing materials, such as papermaking, biofuel, agricultural compost, mushroom cultivation, and waste processing industries.

## Additional file

**Additional file 1: Table S1.** Details of *C. thermocellum* genes used in this study. The cellulosomal genes were amplified from *C. thermocellum* ATCC 27405 genomic DNA using gene-specific primers. **Table S2.** Primers used for the amplification of specific genes from genomic DNA. **Table S3.** Sequencing and qPCR primers used for checking the specific genes. **Figure S1.** Western blotting analysis of CipA using epitope antibody against CipA. (1: negative control, 2: *B subtilis* WB800− cipA + sdbA, 3: *B subtilis* WB800− cipA + sdbA, 4: Marker). **Figure S2.** MASCOT analysis of protein bands.

## Abbreviations

CipA: cellulosome integrative protein A
SdbA: scaffoldin dockerin-binding protein A
Orf2: open reading frame 2
OlpB: outer layer protein B
SLH: S-layer homology
CBM: cellulose-binding module
PASC: phosphoric acid-swollen cellulose
dye-CMC: azo-carboxymethylcellulose
