# Supplementary material for: Biomimetic strategy for constructing Clostridium thermocellum cellulosomal operons in Bacillus subtilis
Source: Biotechnol Biofuels. 2018 Jun 7;11:157. doi: 10.1186/s13068-018-1151-7 (PMC5991470; doi:10.1186/s13068-018-1151-7)

**Additional file 1**

**Biomimetic strategy for constructing *Clostridium thermocellum* cellulosomal operons in *Bacillus subtilis***

Jui-Jen Chang^1,†^, Marimuthu Anandharaj^2,3,4,†^, Cheng-Yu Ho^5^, Kenji Tsuge^6^, Tsung-Yu Tsai^2^, Huei-Mien Ke^2,4^, Yu-Ju Lin^2^, Minh Dung Ha Tran^3,4,5^, Wen-Hsiung Li^2,3,4,7,8,^*, and Chieh-Chen Huang^5,^*

^1^Department of Medical Research, China Medical University Hospital, China Medical University, Taichung 402, Taiwan

^2^Biodiversity Research center, Academia Sinica, Taipei 11529, Taiwan.

^3^Molecular and Biological Agricultural Sciences Program, Taiwan International Graduate Program, National Chung Hsing University and Academia Sinica, Taipei 11529, Taiwan.

^4^Graduate Institute of Biotechnology, National Chung Hsing University, Taichung 40227, Taiwan.

^5^Department of Life Sciences, National Chung Hsing University, Taichung 40227, Taiwan

^6^Institute for Advanced Biosciences, Keio University, 403-1 Nipponkoku, Daihoji, Tsuruoka, Yamagata 997-0017, Japan

^7^Biotechnology Center, National Chung Hsing University, Taichung 40227, Taiwan.

^8^Department of Ecology and Evolution, University of Chicago, Chicago, IL 60637, USA.

†These authors contributed equally to this work.

*Correspondence should be addressed to W-HL ([whli@uchicago.edu](mailto:whli@gate.sinica.edu.tw)) and C-CH ([cchuang@nchu.edu.tw](mailto:cchuang@nchu.edu.tw)).

**Additional file 1**

**Table S1:** Details of *C. thermocellum* genes used in this study. The cellulosomal genes were amplified from C. thermocellum ATCC 27405 genomic DNA using gene-specific primers.

| **Gene** | **Gene description** | **Locus tag** | **Region** | **Length (bp)** | **Protein ID** |
| --- | --- | --- | --- | --- | --- |
| **CipA** | Cellulosomal integrating protein A | Cthe_3077 | 3619516-3625077 | 5579 | ABN54273.1 |
| **SdbA** | Cell-surface anchoring protein | Cthe_1307 | 1586232-1588331 | 2100 | ABN52539.1 |
| **XynZ** | Endo β 1-4 Xylanase (GH10) | Cthe_1963 | 2340386-2342916 | 2531 | ABN53181.1 |
| **XynC** | Endo β 1-4 Xylanase (GH10) | Cthe_1838 | 2181190-2183066 | 1877 | ABN53059.1 |
| **CelS** | Exoglucanase (GH48) | Cthe_2089 | 2482535-2484777 | 2243 | ABN53296.1 |
| **CelK** | Exoglucanase (GH9) | Cthe_0412 | 516236-518940 | 2705 | ABN51650.1 |
| **CelA** | Endoglucanase (GH8) | Cthe_0269 | 331554-333004 | 1451 | ABN51508.1 |
| **CelR** | Endoglucanase (GH9) | Cthe_0578 | 706080-708307 | 2228 | ABN51814.1 |

**Table S2:** Primers used for the amplification of specific genes from genomic DNA

| **Primer** | **Sequence** | **Reference** |
| --- | --- | --- |
| CipA F (Sfi*I*) | TAGGGCCTGTTTGGCCTTTGGGAGGAATGGTAGATGAG | This study |
| CipA R (DraIII) | TAGCACTCAGTGTTACTGTGCGTCGTAATCACTTG | This study |
| CelK F (BalI) | TAGGCCATGACGGCTAACGGGAGGTAGATTTATGAATTTCAG | This study |
| CelK R (BalI) | TAGGCCGTCGTGGCTTATTTATGTGGCAATACATCTATCTCTTTAAGAATATATC | This study |
| CelS F (DraIII) | TAGCACCGAGTGAGGGGAAGATGGAGAGAATG | This study |
| CelS R (DraIII) | TAGCACTGGGTGTTAGTTCTTGTACGGCAATGTATCTATTTC | This study |
| CelR F (DraIII) | TAGCACCCAGTGAAAGGGGGAGATATATAGTGAAAAAACTC | This study |
| CelR R (DraIII) | TAGCACGACGTGTCATGAATTTCCGGGTATGGTTG | This study |
| OlpB F (DraIII) | TAGCACGTCGTGGGGGGAATTTTTTCTTCATGAAACG | This study |
| OlpB R (DraIII) | TAGCACCATGTGTTAATCTTCGAGCAATTTAACGAATTTTTCTTTCTC | This study |
| CelA F (DraIII) | TAGCACATGGTGAAAAGGAGGAAAAAAAAGTGAAGAACGTAAAAAAAAG | This study |
| CelA R (DraIII) | TAGCACCCAGTGCTAATAAGGTAGGTGGGGTATG | This study |
| XynC F (DraIII) | TAGCACTGGGTGTTTTGGGAGGTAGATCTATGCTG | This study |
| XynC R (DraIII) | TAGCACCTAGTGTCAAAGTTCTCTCAGAACGAGTTTTTTC | This study |
| XynZ F (DraIII) | TAGCACTAGGTGCAAAAAGGAGGAGAAACATGTCAAG | This study |
| XynZ R (DraIII) | TAGGGCCAAGAAGGCCTCAATAGCCCATAAGAGCTTCC | This study |

**Table S3:** Sequencing and qPCR primers used for checking the specific genes

| **Primer** | **Sequence** | **Reference** |
| --- | --- | --- |
| sCipA-15 F (69-85) | GCAGACAGTATCGGCGG | This study |
| sCipA-15 R (168-150) | TGGCACTCCTTTCAGGGTT | This study |
| sCipA-30 F (2525-2541) | AAGTAGGCGGATTTGCG | This study |
| sCipA-30 R (2629- 2609) | GTTATGTAGGTGTTGCAGGTT | This study |
| sCipA-60 F (1575- 1595) | TGTAGTACCATCAACACAGCC | This study |
| sCipA-60 R (1698- 1674) | CACCTTAATCTTTATTGCATTCGGA | This study |
| Cel K-2 F (2457-2474) | GCCAGGAGGAGTAGACCC | This study |
| Cel K-2 R (2557-2535) | TATATCTCTTCAATGCCACAGCG | This study |
| Cel K-167 F (1383-1403) | CGGAATGGTACACCACAAAAT | This study |
| Cel K-167 R (1496- 1480) | TTCAAAGTCGCAGCCGT | This study |
| Cel S-49 F (1801-1819) | AACGGTGACAAGATTCAGC | This study |
| Cel S-49 R (1903-1887) | CGCCTCTCAAGTATGCC | This study |
| Cel R-19 F (1245-1264) | TCCTGAATACCACAGACACG | This study |
| Cel R-19 R (1350-1328) | GGCAACCTCATTTGTTACATAGT | This study |
| Cel A-140 F (531-552) | GCAGGAAGCAAGGACATTGATA | This study |
| Cel A-140 R (632-613) | GTTACTGATGAACCTCCCCA | This study |
| Xyn C-213 F (817-833) | AGACCCCTGTTGGAGTT | This study |
| Xyn C-213 R (936-916) | AGCATTTTCGTCCTGAGAGTA | This study |
| Xyn Z-92 F (430-453) | GATTTGCTCAACAGTCTTATTCCC | This study |
| Xyn Z-92 R (538-519) | TAAACGATTGTCCTCCACCC | This study |
| Xyn Z-248 F (1178-1197) | GCAGCATTAGCAAAGTCACC | This study |
| Xyn Z-248 R (1289-1273) | TCCCCCAGACCTGTGGA | This study |
| 00196-195 F (1565-1583) | TTGTTTTCGCTGGTATGCC | This study |
| 00196-195 R (1666-1644) | GAGCCCAAGTGTATGGTAAGTGA | This study |
| SdbA-320 F (1727-1753) | GATGTGTCCGAAAAAGACTACTATTAT | This study |
| SdbA-320 R (1875-1853) | ATCCTGAGAGCATTTGTTGTAAG | This study |
| Bu 16S-77 F (189-207) | ACCGCATGGTTCGAACATA | This study |
| Bu 16S-77 R (493-477) | CCGTCAAGGTGCCGCCC | This study |

**Additional Figures**


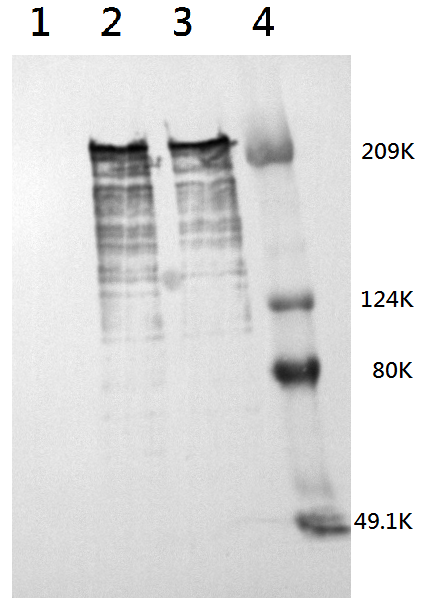


**Figure S1:** Western blotting analysis of CipA using epitope antibody against CipA. (1: negative control, 2: *B subtilis* WB800- cipA + sdbA, 3: *B subtillis* WB800- cipA + sdbA, 4: Marker)

**Figure S2:** MASCOT analysis of protein bands


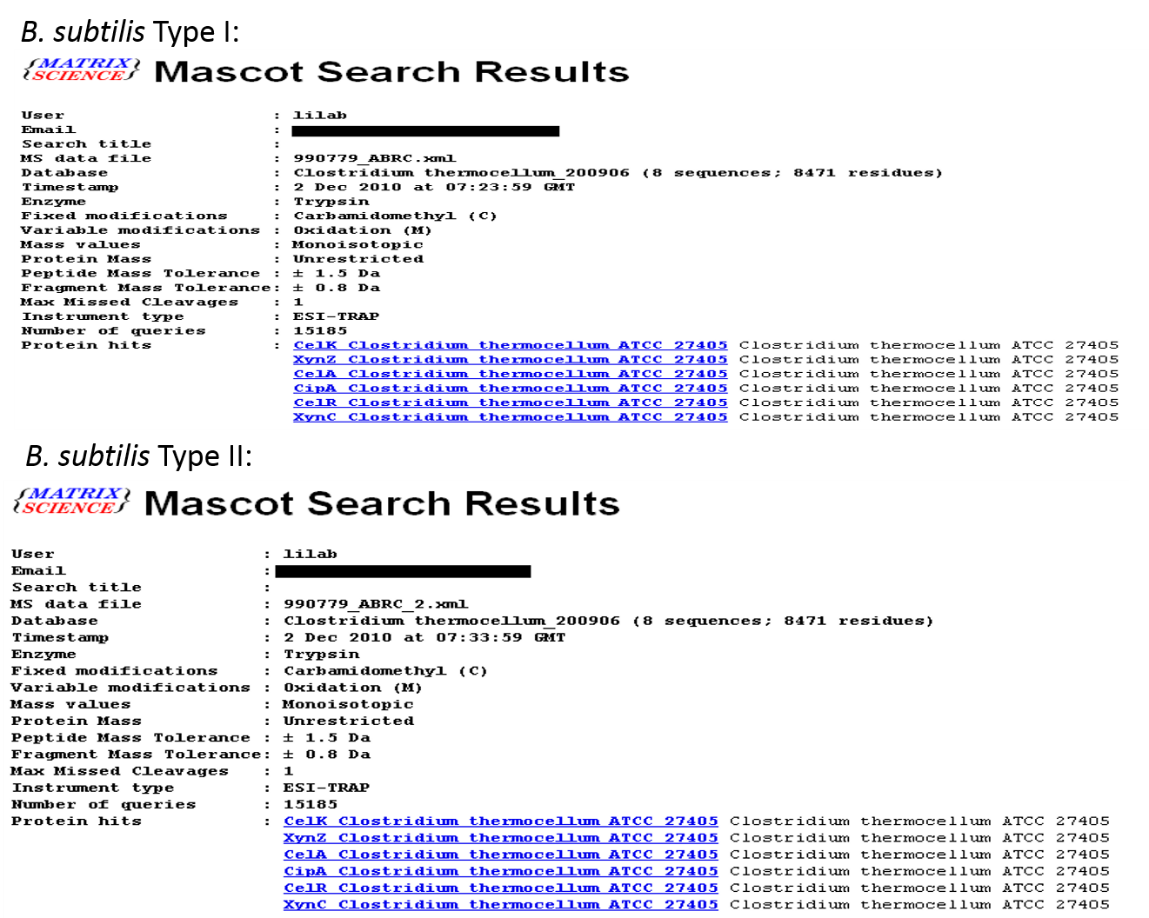

Supplement: Supplementary file 1 — Additional file 1: Table S1. Details of C. thermocellum genes used in this study. The cellulosomal genes were amplified from C. thermocellum ATCC 27405 genomic DNA using gene-specific primers. Table S2. Primers used for the amplification of specific genes from genomic DNA. Table S3. Sequencing and qPCR primers used for checking the specific genes. Figure S1. Western blotting analysis of CipA using epitope antibody against CipA. (1: negative control, 2: B subtilis WB800− cipA + sdbA, 3: B subtilis WB800− cipA + sdbA, 4: Marker). Figure S2. MASCOT analysis of protein bands. [file 13068_2018_1151_MOESM1_ESM.docx]
